# Development and Evaluation of an Electrochemical Biosensor for Detection of Dengue-Specific IgM Antibody in Serum Samples

**DOI:** 10.3390/diagnostics11010033

**Published:** 2020-12-26

**Authors:** Om Parkash, Muhammad Amiruddin Abdullah, Chan Yean Yean, Shamala Devi Sekaran, Rafidah Hanim Shueb

**Affiliations:** 1 Department of Medical Microbiology and Parasitology, School of Medical Science, Universiti Sains Malaysia, Kubang Kerian 16150, Malaysia; oom.parkash@cmc.edu.pk (O.P.); amiruddin@usm.my (M.A.A.); yeancyn@yahoo.com (C.Y.Y.); 2 Department of Pathology, Chandka Medical College, Shaheed Mohatrama Benazir Bhutto Medical University, Larkana 71770, Pakistan; 3 Faculty of Applied Sciences, UCSI University, No. 1 Menara Gading, UCSI Heights, Kuala Lumpur 56000, Malaysia; shamala@ucsiuniversity.edu.my; 4 Institute for Research in Molecular Medicine, Universiti Sains Malaysia, Kubang Kerian 16150, Malaysia

**Keywords:** electrochemical biosensor, dengue IgM antibody, SPCEs, streptavidin/biotin, protein A/G

## Abstract

Dengue is an arbovirus disease transmitted mainly by *Aedes* mosquitoes. As dengue shares similar clinical symptoms with other infectious diseases, prompt and accurate diagnosis is pivotal to clinicians’ decisions on appropriate management. Conventional diagnostic tests to detect the dengue-specific IgM antibody are limited in their performance and ease of use. To address these issues, we developed and evaluated a biosensor based on screen-printed carbon electrodes (SPCEs) for the detection of dengue-specific immunoglobulin M (IgM) antibodies. Various optimisations were performed in order to increase the sensitivity and specificity of the biosensor. For optimal and proper orientation of the paratope sites of goat anti-human IgM capture antibodies (GAHICA), various antibody techniques, including passive, covalent, protein A, protein G and streptavidin/biotin systems, were tested on the SPCEs. The assay reagents for the biosensor were also optimised prior to its evaluation. Analytical sensitivity evaluation was carried out using pooled sera, while analytical specificity evaluation was conducted on a panel of six non-dengue serum samples. Subsequently, diagnostic sensitivity and specificity evaluation were performed using 144 reference samples. Electrochemical current signals generated from H_2_O_2_ catalysed by HRP-labelled anti-dengue detection antibodies were measured using the chronoamperometric technique. With a limit of detection (LOD) of 10^6^ serum dilution, the analytical sensitivity of the developed biosensor was 10 times higher than commercial ELISA. The analytical specificity of this dengue IgM biosensor was 100%. Similarly, the biosensor’s diagnostic performance was 100% for sensitivity, specificity, positive predictive value (PPV) and negative predictive value (NPV). These findings suggest that the developed biosensor has a great potential to be used to diagnose dengue after seroconversion.

## 1. Introduction

Dengue is among the most important human viral diseases that exert a considerable global health burden. In the past few decades, the global prevalence of dengue has risen significantly. Currently, dengue, which poses risk to approximately 3.6 billion people, is endemic in over 100 tropical and subtropical countries. Annually, 390 million dengue cases are estimated to occur. Dengue patients often experience a variety of clinical manifestations, ranging from mild dengue fever to life-threatening severe dengue [1,2]. Although a vaccine is now commercially available, it is not widely accessible in many countries [3]. Furthermore, clinical diagnosis could be challenging, since certain signs and symptoms of dengue overlap with other diseases, such as chikungunya, malaria, typhoid, typhus and leptospirosis [4,5]. Thus, effective management of dengue patients and subsequent outbreak control depend partly on rapid and accurate diagnosis of suspected patients [6,7].

Serological assays to detect the dengue-specific non-structural 1 (NS1) antigen or immunoglobulin M (IgM) antibodies are among the most common diagnostic methods used for the detection of dengue virus infection [8,9]. The most widely used serological assays are the lateral flow test and the enzyme-linked immunosorbent assay (ELISA) [10]. The former test is rapid, taking between 10 to 30 min to complete, but tends to exhibit lower sensitivity and specificity than ELISA [11]. In contrast, ELISA requires expensive equipment for its analysis and automation [12].

Electrochemical biosensors are an emerging technology that could be incorporated into clinical diagnostic kits to enhance diagnosis, since it is highly sensitive, specific, simple, rapid and reliable [13]. Compatibility with new microelectronic tools, minimum energy consumption, cost effectiveness and exclusion from sample turbidity and colour are other advantages of electrochemical biosensors [14,15]. An electrochemical biosensor employing screen printed carbon electrodes (SPCEs) is an attractive alternative to the current diagnostic method, as it demonstrates a sensitivity that is as good as ELISA and a portability comparable to the lateral flow assay. The strong chemical inertness of carbon electrodes provides a variety of working potential with low electrical resistivity. They also have a crystalline structure that provides low residual currents and a high signal to noise ratio [16,17]. In addition, the SPCEs electrochemical biosensor has advantages such as low production cost, simplicity, portability, versatility and ease of mass production.

We previously developed an electrochemical biosensor for the detection of dengue-specific non-structural 1 (NS1) antigen. The biosensor, which was designed based on the principle of direct ELISA and fabricated using SPCEs, exhibited good sensitivity and specificity [18]. However, a more common approach for serological diagnosis of dengue in an endemic country, such as Malaysia, is the simultaneous detection of the dengue NS1 antigen and IgM antibodies. In infected patients, the dengue NS1 biomarker is detectable on the first day of fever. Several days following infection, the dengue NS1 level declines, while dengue-specific IgM and IgG antibodies start to elevate [19]. Thus, concurrent detection of the dengue NS1 antigen and IgM antibodies increases the accuracy and ease of diagnosing dengue at any time-point of infection, averts the drawback of repeated testing and ultimately provides prompt diagnosis to doctors. This study therefore aimed to develop a sensitive electrochemical biosensor for detecting dengue IgM antibodies, with the expectation that, in the future, it could be combined with an NS1 biosensor as a complete test package for dengue. In this proposed IgM electrochemical biosensor, we used an approach similar to our previously developed NS1 biosensor. However, rather than the principle of direct ELISA utilized for the NS1 biosensor, this IgM electrochemical biosensor was constructed based on IgM capture ELISA (MAC-ELISA) [18]. Consequently, the fabrication involved entirely different optimisation procedures and reagents than the previously developed NS1 biosensor. Furthermore, we demonstrated the potential application of the IgM biosensor on clinical samples.

## 2. Materials and Methods

### 2.1. SPCE, Chemicals and Assay Reagents

The SPCEs were bought from Scrint Technology (Penang, Malaysia). The SPCEs were based on three carbon electrodes, namely, the working electrode, counter electrode and reference electrode. The working electrode was used for developing an immunoassay, while the two other electrodes surrounded the working electrode to form a complete electrical circuit. The electrochemical characterisation, reproducibility and stability of the SPCEs have been evaluated in our previous study [18]. Bovine serum albumin (BSA), ethanolamine hydrochloride, streptavidin and GAHICA were purchased from Sigma (St. Louis, MO, USA). BupH MES buffered saline packs were purchased from Thermo Scientific (Rockford, IL, USA). Lightning-link horseradish peroxidase (HRP) and lightning-link biotin were purchased from Innova Biosciences (Cambridge, UK). A recombinant polyvalent dengue antigen was purchased from RayBiotech (Norcross, GA, USA). Anti-dengue virus antibodies were acquired from Abcam (Cambridge, UK). Carboxymethyldextran (CMD) (500,000 MW) was purchased from Fluka (Gillingham, UK). Ready-to-use 3,30,5,50-tetramethylbenzidine (TMB) substrates were purchased from Promega (Madison, WI, USA) and the dengue IgM capture ELISA kit was purchased from Panbio Diagnostics (Brisbane, Australia).

### 2.2. Apparatus

Chronoamperometric measurements were performed with an Autolab PGSTAT III potentiostat/galvanostat (Metrohm Autolab B.V., Utrecht, The Netherlands), which was interfaced to Nova software (version 1.6, Metrohm Autolab B.V., Utrecht, The Netherlands).

### 2.3. Clinical Samples

Blood samples were obtained from suspected dengue patients visiting the clinic or emergency department at Hospital Universiti Sains Malaysia. The collected samples were tested with the dengue IgM capture ELISA (Panbio) at the Department of Medical Microbiology and Parasitology, Universiti Sains Malaysia (USM). Afterwards, ELISA positive and negative samples were pooled separately and used to develop and optimise the electrochemical biosensor. For diagnostic evaluation, 144 reference samples were provided by the Department of Microbiology, Universiti Malaya (UM) (Table 1). This research has been approved by the USM Committee on Human Ethics (USM/JePeM/270.4.(1.3), 09/10/2013).

### 2.4. Immobilisation of GAHICA on SPCEs

A number of methods were evaluated to attain the best immobilisation strategy of GAHICA on the carbon working electrode (CWE). These strategies were either based on random antibody immobilisation (passive adsorption or covalent binding) or oriented antibody immobilisation (employing protein A, protein G or streptavidin-biotin).

#### 2.4.1. Passive Adsorption

For passive adsorption, 20 µL (10 µg/mL) of GAHICA diluted in carbonate buffer (pH = 9.4) was pipetted on CWE. The SPCEs were then incubated overnight at 4 °C. Following washing, the CWE was blocked with 3% BSA for 15 min.

#### 2.4.2. Covalent Immobilization

EDC/NHS, a chemical cross-linker, was used to immobilise GAHICA permanently on CWE of SPCEs. Initially, 20 µL (50 mg/mL) of CMD was added on the CWE to introduce the carboxylic group (COOH). Following overnight incubation, unbound CMD was washed away with PBS. Afterwards, the COOH group was activated on the CWE by incubating 20 µL of the cross-linkers (0.4 M of EDC and 0.1 M of NHS) for 10 min at room temperature. The cross-linker reacts with the COOH group and converts it into an active *O*-acylisourea intermediate, which is readily converted by nucleophilic attack from the primary amino group into an amide bond and isourea as a by-product. The amino group with primary reactive amine side chains on the capture antibodies was then linked by adding 20 µL (10 µg/mL) of GAHICA and incubated for 1 h. 20 µL of 1 M of ethanolamine hydrochloride was subsequently pipetted on the SPCEs for 10 min in the dark to remove unbound ester groups. Later, 3% BSA was added on the CWE for 15 min to block non-specific binding.

#### 2.4.3. Protein A or G Based Immobilization

Protein A or G was immobilised on separate CWE by pipetting 20 µL (5 mg/mL) of each protein and incubating for 24 h. Subsequently, 20 μL (10 μg/mL) of GAHICA was incubated on the CWE for 1 h. Following washing, 20 μL of (3%) BSA was added on CWE for 15 min to block non-specific bindings.

#### 2.4.4. Streptavidin-Biotin Based Immobilization

For a stable immobilization of streptavidin on the CWE, the covalent technique as stated in Section 2.4.2 was used. Following incubation of EDC-NHS, 20 µL (50 µg/mL) of streptavidin was pipetted and incubated for 1 h. Upon washing, 20 µL/mL of biotinylated GAHICA (10 µg/mL) was pipetted on the CWE and incubated for 1 h. Then, 20 µL of 1 M of ethanolamine hydrochloride was pipetted on the CWE and incubated for 10 min in the dark. Afterwards, 20 µL of 3% BSA was added on the CWE for 15 min.

### 2.5. Visualisation of Immobilised GAHICA Using Field Emission Scanning Electron Microscopy (FESEM)

Following antibody immobilisation as described in Section 2.4.4, the scanning electron micrograph images were obtained from FESEM at an acceleration voltage of 10 kV and a working distance of 10 μm.

### 2.6. Fabrication of Electrochemical Biosensor

Following successful immobilisation of GAHICA on the SPCEs, dengue IgM ELISA positive serum samples were pooled and used as a positive control/test, while pooled negative serum samples were used as a negative control. Pooled serum samples were diluted 1:100 in 0.01 M of PBS and then pipetted on the CWE to allow reaction with GAHICA for 1 h. Following incubation and washing, 20 µL (5 µg/mL) of dengue antigen prepared in PBS was added on the CWE. To complete the assay, 20 µL (5 µg/mL) of HRP-conjugated anti-human IgM detection antibodies was added on the CWE and left for 1 h. Before electrochemical measurement, the electrodes were washed with PBS. The cyclic voltammograms analysis following the immobilisation of GAHICA and other assay reagents showed a decrease in redox peaks, similar to that described in our previous study [18], and therefore were not discussed in this study.

### 2.7. Quantification of Electrochemical Signals

To generate an electrochemical response, 70 µL of TMB substrate was pipetted on the three electrodes present on the SPCEs to immerse all three electrodes. Subsequently, electrochemical signals generated from the catalysis of H_2_O_2_ by the HRP enzyme were measured by applying a fixed reduction potential of −200 mV to the reference electrode. A cut-off value was established during each experiment to differentiate positive from negative results, and was obtained by calculating the average of three negative samples plus three times the standard deviation (SD).

### 2.8. Analytical and Diagnostic Evaluation of Dengue IgM Biosensor

For successful commercialisation, it is imperative that the established biosensor satisfies the criteria of a standard diagnostic test, such as exhibiting high sensitivity and specificity. Considering this fact, analytical and diagnostic evaluation was carried out for the developed biosensor.

#### 2.8.1. Determination of Analytical Sensitivity

To determine the analytical sensitivity of the proposed biosensor, the pooled serum sample containing dengue-specific IgM antibodies was diluted from 10^1^–10^7^. These dilutions were later tested on the developed dengue IgM biosensor and a commercially available dengue IgM capture ELISA (Panbio). The biosensor assay was performed as mentioned in Section 2.5, while the ELISA assay was carried out according to the protocol provided by the manufacturer.

#### 2.8.2. Determination of Analytical Specificity

To ensure that the developed dengue IgM biosensor selectively detected dengue IgM antibodies, it was exposed to a panel of different samples containing IgM antibodies specific either for hepatitis B, hepatitis C, chikungunya, leptospirosis, malaria or healthy serum samples. The serum was diluted in 1:100 using PBS buffer.

#### 2.8.3. Determination of Diagnostic Sensitivity and Specificity

Reference samples (Table 1) were tested to assess the diagnostic sensitivity and specificity of the developed dengue IgM biosensor.

### 2.9. Statistical Analysis

Statistical analysis was performed using Statistical Package for the Social Sciences (SPSS) software, version 25. All of the assays were carried out on three independent SPCEs, and the values were presented as mean ± standard error (SE). One-way ANOVA with Tukey’s post hoc test was used for the analysis. A *p*-value ˂ 0.05 was considered statistically significant. Diagnostic sensitivity, specificity, PPV and NPV were calculated using the online MEDCALC^®^ calculator.

## 3. Results

### 3.1. Determination of a Suitable Immobilisation Technique for GAHICA

**Figure 1:**
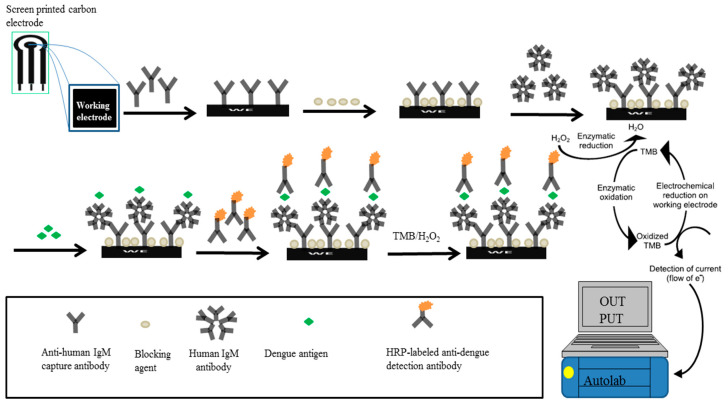
Schematic diagram of the screen printed carbon electrode (SCPE)-based dengue IgM biosensor. The biosensor was constructed by sequentially adding optimised concentration of anti-human IgM capture antibody, blocking agent, human IgM antibody or serum sample, dengue antigen and detection antibody, with washing steps in between. Electrochemical signal was generated following addition of TMB substrate.

**Figure 2:**
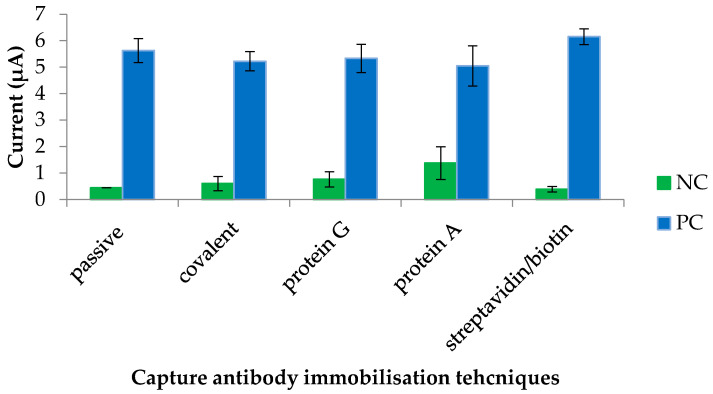
Comparison of various immobilisation techniques for the goat anti-human IgM capture antibody. NC: negative control consisting of a dengue IgM negative serum sample; PC: dengue IgM positive serum sample.

**Figure 3:**
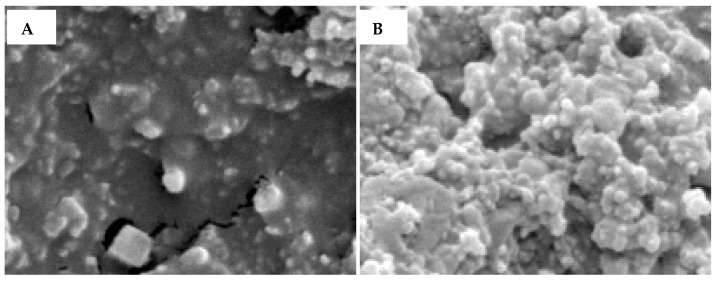
FESEM surface images of (**A**) a bare carbon electrode and (**B**) a carbon electrode modified with an anti-human IgM antibody using the streptavidin/biotin immobilisation system.

The workflow of the dengue IgM biosensor is shown in Figure 1. In this study, investigation of appropriate antibody immobilisation techniques for the GAHICA was performed using random and oriented antibody immobilisation techniques (passive, covalent, protein A, protein G and streptavidin/biotin). Electrochemical responses ensuing from the various immobilisation methods are shown in Figure 2. All of the antibody immobilisation techniques demonstrated stability throughout the duration of the detection assay, as the capture antibody was always present to bind the dengue antigen, resulting in generation of current signals. There was no significant difference (*p >* 0.05) in the current signals generated from the various immobilisation techniques assessed in this study. However, covalent, protein A and protein G techniques generated slightly lower current signals. Interestingly, compared to these techniques, passive immobilisation produced a higher current response with low background signals. However, the best antibody immobilisation that yielded maximum current signals with minimum background signals (0.31 µA) was observed following the use of the streptavidin/biotin immobilisation system. The successful GAHICA immobilisation using this technique was also evident from FESEM images (Figure 3). The surface of CWE with immobilised GAHICA showed cloudy clusters when compared to blank CWE. Therefore, this technique was selected for immobilising the GAHICA on the CWE.

### 3.2. Optimisation of Assay Reagents

**Figure 4:**
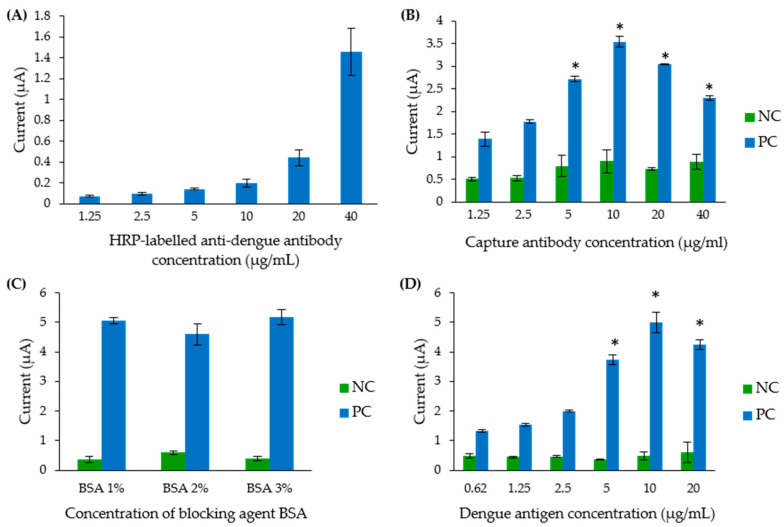
Optimisation of the concentration of various assay reagents. (**A**) Optimisation of the detection antibody concentration. (**B**) Optimisation of the goat anti-human IgM antibody. (**C**) Optimisation of the BSA concentration for blocking the electrode’s surface. (**D**) Optimisation of the dengue antigen concentration. NC: negative control (consisting of a dengue IgM negative serum pool), PC: positive control (consisting of a dengue IgM positive serum pool). * Indicates statistically significant current signals.

The concentration of various assay reagents used in the electrochemical biosensor assay was optimised to further enhance the electrochemical response. To simultaneously reduce background signals and increase current signals, various parameters of the biosensor were analysed. Optimum concentration of the detection antibody was determined. CWE was first blocked with 20 µL of 3% BSA for 15 min. Then, different concentrations (ranging from 0 to 40 µg/mL) of the HRP-labelled anti-dengue detection antibody were incubated on the CWE. Following washing, the electrochemical response was measured. As demonstrated in Figure 4A, a significant background current signal was not observed until the concentration of the HRP-conjugated anti-dengue detection antibody used was 10 µg/mL or higher. Consequently, to reduce background signal, 5 µg/mL of detection antibody was utilised in the succeeding assays.

Secondly, the optimum concentration of GAHICA was determined by testing various concentrations ranging from 1.25 to 40 µg/mL. As shown in Figure 4B, a significant current response was observed when 5, 10, 20 and 40 µg/mL GAHICA were used (*p* < 0.05), although the current signals directly correlated with the concentration of the capture antibody only in the presence of 1.25 to 10 µg/mL of the antibody. The maximum current response (3.54 µA) was generated when 10 µg/mL of the capture antibody was utilised.

To further reduce background signal, 1%, 2% and 3% BSA were tested in this dengue IgM biosensor assay (Figure 4C). Interestingly, a similar response with no significant difference in current signals (*p* > 0.05) was observed when the non-specific reaction in the assay was blocked using these different BSA concentrations. Subsequently, 3% BSA was chosen in the following experiment, as it generated the highest current signal.

The sensitivity of the developed IgM biosensor could be further boosted by establishing a suitable concentration of the polyvalent dengue antigen. As shown in Figure 4D, the current signal demonstrated an increasing trend when a concentration of up to 10 µg/mL of the dengue antigen was added during the assay. In contrast, the addition of 20 µg/mL of the dengue antigen caused a slight decrease in the current signal. Therefore, a 10 µg/mL concentration of the polyvalent dengue antigen was chosen for further experiments.

### 3.3. Analytical Sensitivity and Specificity of the Dengue IgM Biosensor

**Figure 5:**
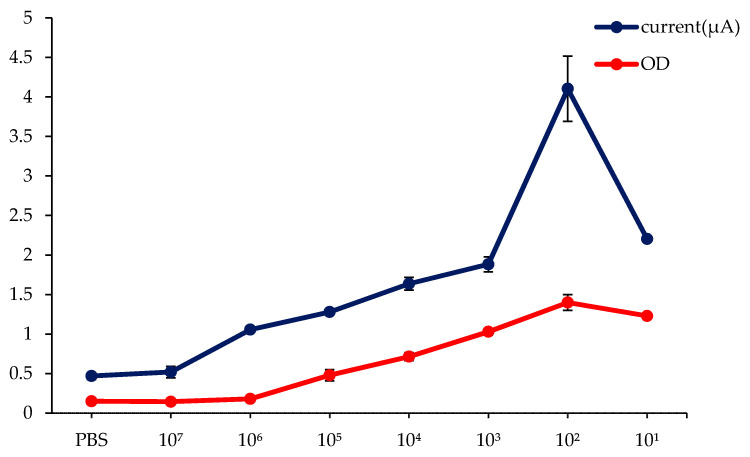
Comparative analysis of the analytical sensitivity of the dengue IgM biosensor against commercial dengue IgM ELISA. NC: negative control (consisting of a dengue IgM negative serum pool). PBS was used as diluent for the serum.

Electrochemical responses produced from different dilutions of serum samples are shown in Figure 5. When tested on the biosensor, serum dilutions of 10^1^ to 10^6^ generated current signals above the cut-off value (0.59 µA). Hence, the LOD of the developed biosensor was 10^6^, and a linear relationship with the current signals was within 10^3^ to 10^6^ serum dilutions. However, as shown in Figure 5, the peak current response (4.10 µA) generated was at a 10^2^ dilution, suggesting the ideal dilution for the assay. The electrochemical response was slightly inhibited at low serum dilution (10^1^). This could be due to the matrix effect, where, at lower dilution, other substances present in the serum affect the binding between the target and its respective antibodies, resulting in a lower current signal. Further serum dilution can, however, weaken this matrix effect.

As a comparison, the analytical sensitivity evaluation for commercial ELISA was also carried out using the same serum dilutions. The cut-off value for the ELISA technique was found to be 0.43 optical density (OD). In contrast to the dengue IgM biosensor, only serum dilution in the range of 10^1^ to 10^5^ (Figure 5) had an OD above the cut-off value. Thus, the LOD of ELISA was 10^5^ serum dilution, which was 10 times less sensitive than the developed dengue IgM biosensor.

**Figure 6:**
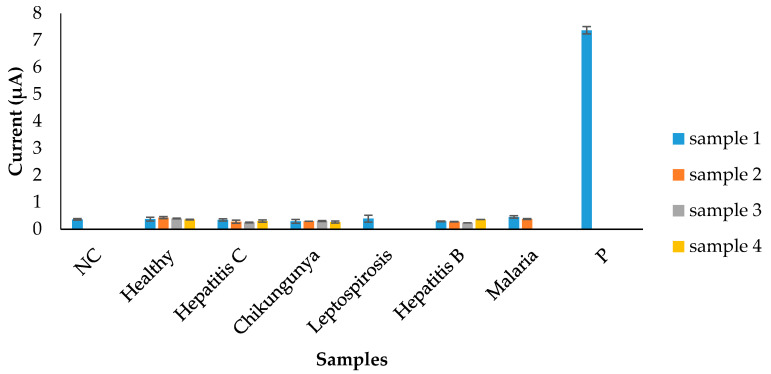
Analytical specificity evaluation of the electrochemical biosensor using serum samples from different infections. NC: background control against dengue IgM negative serum pool, P: positive detection against target dengue IgM positive serum pool.

The electrochemical response generated during analytical specificity evaluation is demonstrated in Figure 6. A cut-off value of 0.45 µA was obtained from triplicates of dengue negative samples. When the developed biosensor was tested with a panel of six non-dengue serum samples, all of the serum samples generated current signals below the cut-off value. On the other hand, the developed biosensor generated a maximum current response (7.37 µA) for the dengue-specific IgM positive pooled serum sample (Figure 6). This suggests that the developed biosensor was highly selective for dengue-specific IgM antibodies, as it could clearly distinguish dengue IgM positive samples from the non-dengue sera.

### 3.4. Diagnostic Sensitivity and Specificity Evaluation of the Dengue IgM Biosensor Using Real Serum Samples

**Figure 7:**
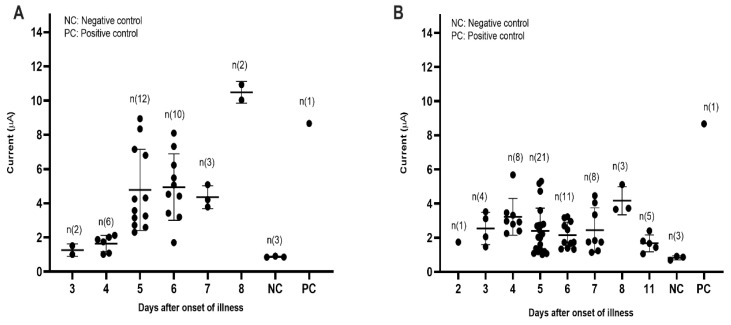
Diagnostic evaluation of the dengue IgM electrochemical biosensor using dengue positive sera. (**A**) The electrochemical response generated from sera with primary dengue. (**B**) The electrochemical response generated from sera with secondary dengue.

The electrochemical responses of 96 dengue IgM positive samples were classified according to infection status and days after onset of symptoms (Figure 7). As expected, the developed dengue IgM biosensor could accurately detect all dengue IgM positive samples from both primary (Figure 7A) and secondary (Figure 7B) infections, with current signals above the cut-off value (0.67 µA). The highest (4.94 µA) and lowest (1.26 µA) average current signals were attained from samples collected on day six and day three post-onset of symptoms, respectively, during primary dengue. Markedly, in comparison with secondary dengue, the average current signals obtained from sera with primary dengue were higher from day five post-infection.

**Figure 8:**
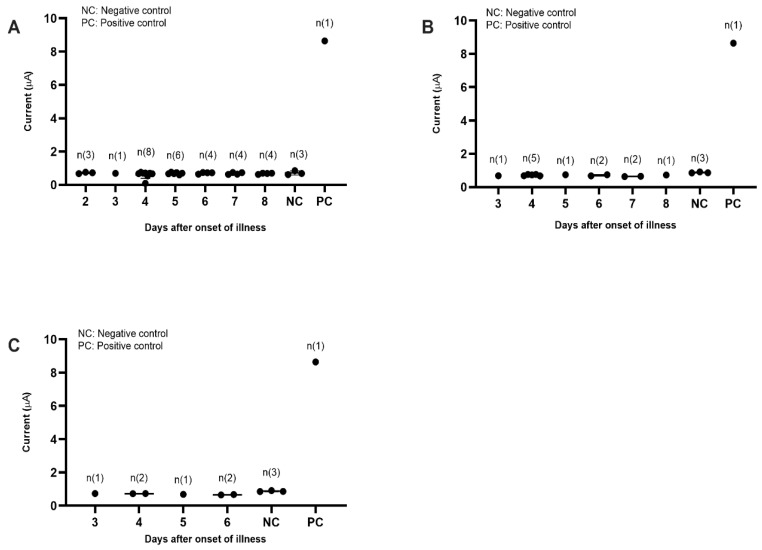
Diagnostic evaluation of the dengue IgM electrochemical biosensor using dengue negative sera. (**A**) The electrochemical response generated against dengue IgM and NS1 negative samples. (**B**) The electrochemical response generated against dengue IgM negative but NS1 positive samples (primary dengue). (**C**) The electrochemical response generated against dengue IgM negative samples (secondary infection).

On the other hand, dengue-specific IgM negative samples were used to determine the diagnostic specificity of the biosensor. The profile of electrochemical responses generated from a total of 48 dengue negative serum samples consisting of both dengue IgM and NS1 negative samples, dengue IgM negative but NS1 positive samples (primary infection) and dengue IgM negative samples (secondary infection) are shown in Figure 8A–C, respectively. All of the samples tested using the electrochemical biosensor produced electrochemical signals below the cut-off value (0.87 µA), implying that the developed assay was highly specific for only its target analyte, the dengue-specific IgM antibody.

The developed dengue IgM biosensor had 100% sensitivity, specificity, PPV and NPV (Table 2). The biosensor successfully distinguished 96 dengue-specific IgM antibody positive samples and 48 IgM negative reference samples without any false negative or false positive outcomes.

## 4. Discussion

In this study, a highly specific dengue IgM biosensor was constructed and subsequently assessed for its future potential as an alternative diagnostic assay to confirm dengue infection. For the IgM-based biosensor, proper immobilisation of GAHICA on the CWE of SPCEs is a crucial step to ensure that a sensitive test is developed. This is because the sensitivity of the developed biosensor partly depends on the ability of the GAHICA to successfully bind the CWE with proper orientation of the antibodies. Among the techniques tested, covalent, protein A and protein G techniques generated slightly lower current signals. The lower current response observed during covalent based antibody immobilisation may be due to the effect of antibody crosslinking. The rather harsh treatment of cross-linkers and goat anti-human IgM antibodies could affect the biological activity of the antibodies [20]. Similar to the finding in our previously developed dengue NS1 biosensor [18], the low current signal obtained from the protein A and G immobilisation strategies could be due to the proteins’ weak/low binding affinities towards human IgM and IgA antibodies [21]. In contrast, biotin is known to have strong affinities (ranging from 2.1 × 10^−3^ to 1.7 × 10^−4^ mol/L) toward IgM antibodies [22]. Thus, with the aid of biotin, capture antibodies can be immobilised strongly and with good orientation on the carbon surface. Optimising the various assay reagents used in a biosensor assay is also pivotal to achieving the desired outcomes. Randomly selected concentrations may cause steric hindrance due to an inappropriate ratio of antigen and antibody, and could affect electrochemical responses. Hence, coupled with the use of optimised assay reagents, the performance of the developed IgM biosensor was generally superior to that of the conventional ELISA method.

High sensitivity is always a mandatory criterion for good diagnostic kits. This helps to address, particularly, the concern of a low concentration of antigen/antibodies in some clinical specimens. In terms of analytical sensitivity, the biosensor developed in this study surpassed the commercial dengue ELISA assay. In the future, this notable sensitivity can be further enhanced, perhaps by using a highly efficient electrochemical conductor, such as a gold electrode. Wong et al., 2014 and Atias et al., 2009 used an optical transduction approach for detecting dengue IgM antibodies [2,8]. The approach showed an analytical sensitivity similar to that found in this study. However, compared to optical transduction, a biosensor that employs electrochemical transduction can be easily developed as a point of care (POC) test when combined with a portable reader and integrated with a smartphone to deliver the results. Thus, the developed dengue IgM biosensor provides an appealing alternative POC test that incorporates the sensitivity and specificity of the ELISA assay and the portability of a rapid test.

Specificity is another critical component of a diagnostic assay. Cross-reactivity, a phenomenon that involves the interaction of specific antibodies with more than one antigen/agent, often leads to false positive outcomes in immunoassays, and could affect patient management. Thus, the specificity of an immunoassay typically lies in the quality of the antibodies used for the assay [23]. In this study, the electrochemical biosensor selectively detected dengue-specific IgM antibody positive samples from other nonspecific pathogens. This high specificity of the IgM biosensor is attributable to the selective nature of the antibodies used, as well as the ELISA technique adopted for the biosensor. Similar levels of sensitivity and specificity demonstrated during the diagnostic evaluation of the biosensor suggest its future potential application in the diagnostic laboratory for the diagnosis of dengue in patients.

The IgM antibody is detectable in only 50% of patients from day 3–4 after the onset of illness, although this detection rate increases to 80% by day five and 99% by day 10 onwards [24]. Therefore, serological diagnosis of the dengue IgM antibody is a less reliable test for acute dengue infection. Thus, to increase the detection rate of dengue, irrespective of the disease stage, the developed IgM biosensor should be combined with our previously developed NS1 biosensor [16] for a concurrent detection of both biomarkers.

Notably, some of the reference samples collected on day five, six, seven and eight after the onset of illness were positive for the NS1 antigen and negative for the dengue IgM antibody. Interestingly, the electrochemical biosensor accurately recognized these samples as dengue IgM antibody negative samples. Theoretically, antibody titre is detectable from day five after the onset of fever. In this case, it is possible that these samples were not stored at the optimal temperature, thus, the structure of IgM antibodies may have been compromised. It is also possible that the samples might contain inhibitors that interfere with the antibody/antigen complex.

The developed dengue IgM biosensor demonstrated good analytical sensitivity and specificity, as well as diagnostic sensitivity and specificity. It is therefore a promising tool for the diagnosis of dengue infection after seroconversion. However, the diagnostic evaluation in this study was carried out using a limited number of reference samples. More reference samples, particularly from other closely related flaviviruses, including Japanese encephalitis virus and Zika virus, should be included in the future for a more robust validation of the dengue IgM biosensor.

## 5. Conclusions

In conclusion, an electrochemical biosensor based on the MAC-ELISA principle was successfully developed on screen printed carbon electrodes for the detection of dengue IgM antibodies. The developed biosensor demonstrated a high specificity as well as a high sensitivity with low LOD compared to commercial ELISA. Furthermore, diagnostic evaluation showed that the biosensor could be used for the detection of dengue IgM antibodies in real clinical serum samples. Therefore, the developed electrochemical biosensor provides a good alternative for rapid, sensitive and specific detection of dengue IgM antibodies in real serum samples. However, to decentralize the assay, the autolab reader utilised could be replaced in the future with a portable electrochemical reader or smartphone. Additionally, re-designing the developed biosensor as a multiplex assay with reduced operation time to diagnose different infections could enhance the usability, efficiency, and cost effectiveness of the biosensor in the future.

## Figures and Tables

**Table 1:** Details of reference serum samples used during diagnostic evaluation.

Samples		Quantity
Negative samples	Dengue IgM antibody and NS1 antigen negative	30
	Dengue IgM antibody negative but NS1 antigen positive	18
Positive samples	Dengue IgM antibody and NS1 antigens positive	77
	Dengue IgM antibody positive but NS1 antigen negative	19

**Table 2:** Summary of diagnostic evaluation of the dengue IgM electrochemical biosensor using real serum samples.

Electrochemical Biosensor Results	Diagnosed with Conventional Techniques		Total
	Positive	Negative	
**Positive**	96	0	96
**Negative**	0	48	48
**Total**	96	48	144

## Data Availability

No new data were created or analyzed in this study. Data sharing is not applicable to this article
